# Dental Hygiene

**Published:** 1845-03

**Authors:** 


### Miscellaneous Notices.

*Dental Hygiene*—It is much to be regretted, that while dental therapeutics and surgery, as well as mechanical dentistry, are now cultivated in almost every part of the civilized world, with the most commendable and persevering ardor, the hygiene of the teeth is almost wholly neglected. The old adage, that "an ounce of prevention is better than a pound of cure," will apply with as much force to the dental apparatus as to any other part of the body, and certainly there is no part of the physical organization of man, to which the preventives of disease can be more successfully or effectually applied, than to these organs. Although their diseases can, in almost every instance, during their early or incipient stages, be arrested, how much better would it be to prevent their occurrence? We know it may be said, the dentist is not often consulted, until after the actual occurrence of disease, and oftentimes not until it has progressed so far as to set at defiance all the resources of his art, even to stay its farther progress. Still, he might, by recommending and urging upon the heads of families in which he exercises the duties of his profession, the constant and regular observance of proper hygienic means, do more good, than by staying the ravages of disease, after the dental organism had become actually invaded by it. That it can be done, in the majority of cases, there is not a doubt, and we should be gratified to see the attention of the profession directed to this subject, more than it has hitherto been, or is even at this time.

The plan proposed by the American Society of Dental Surgeons, in the diffusion of correct information upon this important subject, by the distribution of popular tracts, is well calculated to promote this object, and if the members of the profession generally, would co-operate, by assisting to carry out this plan, a vast amount of good might be accomplished.

The hygienic treatment, recommended by Dr. L. S. Parmly, for the teeth, is the most successful that has ever been instituted. It consists in cleaning the teeth regularly four or five times a day with waxed floss silk. It is only by this means that the approximal surfaces of the teeth can be reached, and the foreign matter and vitiated mucus of the mouth removed from between them. Every dentist should be provided with an abundant supply of this article, prepared expressly for the purpose, and he should furnish every one of his patients with it, and such other means as may be necessary to enable him to keep his teeth thoroughly clean.

The paper read by Dr. Parmly, before the American Society of Dental

Surgeons, at its second annual meeting, and published in the second volume of the American Journal of Dental Science, contains much valuable information, and we would again call the attention of the profession to it. We are free to acknowledge that we have been much profited by it, and we doubt not, that others have been also.—*Balt. Ed.*

